# Peri- and Postoperative Complications in Abdominal, Vaginal Extraperitoneal, and Vaginal Intraperitoneal Colpopexy

**DOI:** 10.7759/cureus.81112

**Published:** 2025-03-24

**Authors:** Kimberly C Lince, Devki Patel, Vaishnavi J Patel, Young Son, Virgil DeMario, Sara Sar, David Sussman

**Affiliations:** 1 Department of Clinically Applied Science Education, University of the Incarnate Word School of Osteopathic Medicine, San Antonio, USA; 2 Office of Research and Innovation, Texas Tech University Health Sciences Center Paul L. Foster School of Medicine, Lubbock, USA; 3 Office of Research and Innovation, University of the Incarnate Word School of Osteopathic Medicine, San Antonio, USA; 4 Department of Urology, Jefferson Stratford Hospital, Stratford, USA; 5 Department of Clinical and Applied Science Education, University of the Incarnate Word School of Osteopathic Medicine, San Antonio, USA; 6 Office of Research, Philadelphia College of Osteopathic Medicine, Philadelphia, USA; 7 Department of Urology, Jefferson Washington Township Hospital, Sewell, USA

**Keywords:** abdominal colpopexy, abdominal sacro colpopexy, acs-nsqip, colpopexy, national surgical quality improvement program (nsqip), nsqip, surgical complication, surgical outcome, surgical techniques, vaginal colpopexy

## Abstract

Introduction

Pelvic organ prolapse (POP) is a very common concern for women that can often necessitate surgical intervention, including sacral colpopexy. There are multiple surgical approaches, including vaginal, extraperitoneal, and intraperitoneal. This study aims to identify predictors of the outcomes with the different surgical approaches.

Methods

This retrospective study utilized data from the American College of Surgeons National Surgical Quality Improvement Program (NSQIP) gynecologic-specific database for those who underwent sacral colpopexy for POP. The group was subdivided into surgical approaches that included abdominal, vaginal extraperitoneal, and vaginal intraperitoneal. ANOVA analysis was performed between the three groups, and a multivariate logistic regression was performed to determine the 30-day complication rate.

Results

Among the 1,275 cases analyzed, 326 (25.6%) utilized an abdominal approach, 425 (33.3%) utilized a vaginal approach, and 524 (41.1%) utilized an extraperitoneal approach. The mean age was significantly higher for patients undergoing a vaginal extraperitoneal (64.5 years) compared to abdominal (62.1 years) and vaginal intraperitoneal (61.6 years). There was no difference in the 30-day complication rate between the surgical approaches on adjusted analysis; however, the vaginal extraperitoneal approach had the longest hospital stay, days from operation to discharge, and total operation time.

Conclusion

A variety of surgical approaches for sacral colpopexy can be employed. In our study, we show that the 30-day complication rate was similar between the three approaches; however, the complications were only significant with the abdominal approach showing an increased occurrence of bleeding transfusions when compared to the extraperitoneal approach.

## Introduction

Sacral colpopexy or sacrocolpopexy is a surgical approach utilized for the purpose of repairing apical pelvic organ prolapse (POP). POP occurs in approximately 40% of women [1], with 7-19% of women undergoing a surgical repair [2]. As their condition evolves, these women may begin to experience symptomatic POP, leading to uncomfortable pressure during physical activity, dyspareunia, lower back pain, and/or vaginal spotting [3]. The number of American women with at least one pelvic floor disorder is projected to increase from 28.1 million in 2010 to 43.8 million in 2050. From these data, the number of women with POP will increase by 46% from 3.3 to 4.9 million [4]. There is a 13-20% lifetime risk that patients with symptomatic POP will need surgical repair of their vaginal support defects [5]. There are multiple techniques through which a sacrocolpopexy can be achieved, which include vaginal extraperitoneal, vaginal intraperitoneal, or abdominally. The current gold standard for surgical repair of POP is done via the use of a synthetic mesh to lift the organs, otherwise known as a sacrocolpopexy [6], which allows relief of POP-related symptoms in women who desire to maintain their sexual function [5]. Although many different approaches exist, there are continued gaps in knowledge unaddressed regarding the complications of the different surgical approaches. The POP prevalence rate is rising, and there is minimal data, demonstrating the differences in complication rates between the abdominal, vaginal extraperitoneal, and vaginal intraperitoneal approach of the sacrocolpopexy procedure [7]. The aim of this paper is to analyze the predictors of complications in these three techniques in order to gain a comprehensive understanding of the factors that contribute to complications both perioperatively and postoperatively and to highlight factors that may avoid these complications.

## Materials and methods

Source of data

The American College of Surgeons National Surgical Quality Improvement Program (ACS NSQIP) is a database that adheres to the regulations set forth by the Health Insurance Portability and Accountability Act (HIPAA), and it contains information on patient cases from multiple hospitals in the nation. The principal operative procedure cases are identified based on Current Procedural Terminology (CPT) codes. The aim of the program is to evaluate the quality of care provided after surgical procedures. Since the ACS NSQIP is de-identified, this retrospective study was considered exempt from the institutional review board.

Population of study and analysis

We conducted an analysis of the NSQIP database from 2017 to 2020 to study patients who had undergone an abdominal, vaginal extraperitoneal, or vaginal intraperitoneal colpopexy. To differentiate between types of colpopexy, we used CPT codes for Colpopexy abdominal approach (CPT 57280), colpopexy vaginal extraperitoneal approach (CPT 57282), and colpopexy vaginal intraperitoneal approach (CPT 57283). Therefore, we established three patient cohorts based on the location of colpopexy.

Patient demographic and clinical characteristics were collected; these included age, body mass index (BMI), race, smoking status, American Society of Anesthesiologists (ASA) class, and history of diabetes, hypertension requiring medication, congestive heart failure (CHF), severe chronic obstructive pulmonary disease (COPD), and bleeding disorders. The primary outcome was the composite rate of adverse events within 30 days of the surgery. Adverse events included superficial surgical site infection (SSI), deep incisional SSI, organ space SSI, blood transfusion, sepsis or septic shock, pneumonia, renal failure, myocardial infarction, pulmonary embolism, deep vein thrombosis, cerebral vascular accident or stroke, or unplanned reoperation. A prolonged operation time or length of stay was defined as any value above the mean.

Minor complications included urinary tract infections, superficial SSI, and transfusion. Major complications included unplanned reoperation, unplanned readmission, hospitalization longer than 30 days, deep SSI, organ space SSI, pneumonia, unplanned intubation, pulmonary embolism, unplanned intubation, ventilator longer than 48 hours, progressive renal insufficiency, acute renal failure, stroke or CVA with neurological deficit, cardiac arrest requiring CPR, myocardial infarction, DVT or thrombosis requiring therapy, sepsis, septic shock, wound disruption, and C. difficile colitis. These categories were based on those in a study by Hong et al. and clinical expertise [8].

Comparisons between the abdominal, vaginal extraperitoneal, and vaginal intraperitoneal colpopexy groups were performed as follows: For numeric variables, analysis of variance (ANOVA) was employed. For categorical variables, both the chi-square test and Fisher's exact test were utilized. To identify variables for the logistic regression model, we used the random forest algorithm to generate independent variables of importance. Covariates were then selected upon the random forest model, and multivariate logistic regression was performed. Adjusted analysis was performed with peri- and postoperative complications as dependent variables. Then, a 30-day composite adjusted multivariate regression was performed to compare the three surgical procedures. For variables with complete separation that were unable to be fitted with a traditional logistic regression model, we employed Firth's penalized likelihood method for logistic regression. We conducted the statistical analysis using R version 4.0.2 (R Foundation for Statistical Computing, Vienna, Austria), and statistical significance was accepted at a p-value < 0.05.

## Results

Our study included 1,275 cases, of which 326 (25.6%) utilized an abdominal approach, 524 (41.1%) a vaginal extraperitoneal approach, and 425 (33.3%) a vaginal intraperitoneal approach. We then compared peri- and postoperative complications based on patient demographics. Patients that underwent the vaginal extraperitoneal approach were older (mean age: 64.5 years) and demonstrated significance in complications when comparing the abdominal and intraperitoneal approaches (p values = 0.00126 and <0.001, respectively) (Table 1). Regarding race, Caucasian and Hispanic patients similarly demonstrated a significance in complications when comparing abdominal to extraperitoneal and intraperitoneal approaches (Caucasian patients: p-value = 0.0252 and 0.00534, respectively; Hispanic patients: p-value = <0.001 and 0.00374, respectively). Asian patients differed, showing significance in abdominal vs. intraperitoneal (p-value = <0.001) as well, but also in the extraperitoneal vs. intraperitoneal methods (p-value = 0.00297). The estimated probability of both mortality and morbidity was found to be higher in the patients who underwent the abdominal procedure (0.0013301). Prior abdominal operations were only significant when comparing the intraperitoneal and extraperitoneal methods (p-value = 0.00241). In contrast, prior pelvic operations were significant in abdominal vs. intraperitoneal (p-value = <0.001) and intraperitoneal vs. extraperitoneal (p-value = <0.001). The vaginal extraperitoneal approach was more commonly used among patients with a history of prior pelvic operations (78.6%) and prior abdominal operations (38.0%), while patients with a history of endometriosis more commonly underwent the vaginal intraperitoneal approach (1.6%, p = 0.006).

**Table 1:** Patient demographics Numerical variables have the mean outside the parenthesis and the standard deviation within the parenthesis. Categorical variables are expressed as their number, followed by the percentage within the parenthesis. For numeric variables, analysis of variance (ANOVA) was employed. For categorical variables, both the chi-square test and Fisher's exact test were utilized.

Patient Demographics	Abdominal (N=326)	Extraperitoneal (N=524)	Intraperitoneal (N=425)	Total Cohort (N=1275)	P-value (Abdominal vs Extraperitoneal)	P-value (Abdominal vs Intraperitoneal)	P-value (Extraperitoneal vs Intraperitoneal)	P-value (all three)
Age; mean (SD)	62.098 (10.736)	64.532 (10.619)	61.567 (12.048)	62.922 (11.219)	0.00126	0.531	<0.001	<0.001
Height (in); mean (SD)	63.469 (2.7351)	62.845 (2.7517)	63.045 (2.7970)	63.072 (2.7717)	0.00134	0.0385	0.272	0.00588
Weight (lbs); mean (SD)	161.57 (37.041)	164.68 (36.103)	162.67 (36.849)	163.21 (36.588)	0.227	0.685	0.399	0.452
BMI, mean (SD)	28.144 (6.0116)	29.348 (6.3404)	28.704 (5.9289)	28.826 (6.1366)	0.00617	0.203	0.11	0.0182
Race								
Caucasian	187 (57.4%)	342 (65.3%)	287 (67.5%)	816 (64.0%)	0.0252	0.00534	0.507	0.0117
Hispanic	17 (5.2%)	68 (13.0%)	49 (11.5%)	134 (10.5%)	<0.001	0.00374	0.565	0.00113
American Indian/Alaska Native	1 (0.3%)	5 (1.0%)	7 (1.6%)	13 (1.0%)	0.415	0.147	0.511	0.184
African American	12 (3.7%)	25 (4.8%)	25 (5.9%)	62 (4.9%)	0.559	0.226	0.538	0.377
Asian	2 (0.6%)	12 (2.3%)	27 (6.4%)	41 (3.2%)	0.112	<0.001	0.00297	<0.001
Unknown/Not Reported	123 (37.7%)	138 (26.3%)	77 (18.1%)	338 (26.5%)	<0.001	<0.001	0.00339	<0.001
Other	1 (0.3%)	0 (0%)	0 (0%)	1 (0.1%)	0.384	0.434	-	0.256
Current Smoker	25 (7.7%)	36 (6.9%)	30 (7.1%)	91 (7.1%)	0.763	0.86	1	0.905
Children								
Nulliparity	31 (9.5%)	64 (12.2%)	50 (11.8%)	145 (11.4%)	0.269	0.385	0.911	0.459
Uniparity	39 (12.0%)	56 (10.7%)	58 (13.6%)	153 (12.0%)	0.644	0.567	0.196	0.378
Multiparity	256 (78.5%)	404 (77.1%)	312 (73.4%)	972 (76.2%)	0.688	0.125	0.216	0.22
Estimated Probability of Mortality	0.0013301 (0.0015181)	0.00073400 (0.00081091)	0.00062487 (0.00086031)	0.00085004 (0.0010889)	<0.001	<0.001	0.0452	<0.001
Estimated Probability of Morbidity	0.063557 (0.019234)	0.044138 (0.017434)	0.058189 (0.021249)	0.053787 (0.020943)	<0.001	<0.001	<0.001	<0.001
Insulin-Dependent DM	6 (1.8%)	14 (2.7%)	11 (2.6%)	31 (2.4%)	0.586	0.663	1	0.722
Non-insulin Dependent DM	36 (11.0%)	56 (10.7%)	41 (9.6%)	133 (10.4%)	0.961	0.614	0.676	0.8
Hx of Severe COPD	7 (2.1%)	13 (2.5%)	11 (2.6%)	31 (2.4%)	0.937	0.88	1	0.923
Congestive Heart Failure (CHF) 30 Days Before Surgery	1 (0.3%)	1 (0.2%)	0 (0%)	2 (0.2%)	1	0.434	1	0.726
Hypertension Requiring Medication	126 (38.7%)	232 (44.3%)	181 (42.6%)	539 (42.3%)	0.123	0.311	0.649	0.268
On Dialysis	0 (0%)	1 (0.2%)	1 (0.2%)	2 (0.2%)	1	1	1	0.698
Bleeding Disorders	0 (0%)	3 (0.6%)	3 (0.7%)	6 (0.5%)	0.29	0.262	1	0.394
Open Wound/Wound Infection	0 (0%)	2 (0.4%)	1 (0.2%)	3 (0.2%)	0.527	1	1	0.789
Disseminated Cancer	1 (0.3%)	0 (0%)	1 (0.2%)	2 (0.2%)	0.384	1	0.448	0.515
Ascites	0 (0%)	0 (0%)	1 (0.2%)	1 (0.1%)	-	1	0.448	0.589
Steroid Use for Chronic Condition	6 (1.8%)	10 (1.9%)	5 (1.2%)	21 (1.6%)	1	0.657	0.524	0.645
Prior Abdominal Operations	113 (34.7%)	199 (38.0%)	125 (29.4%)	437 (34.3%)	0.367	0.0751	0.00241	0.00444
Prior Pelvic Operations	247 (75.8%)	412 (78.6%)	240 (56.5%)	899 (70.5%)	0.375	<0.001	<0.001	<0.001
Endometriosis	5 (1.5%)	2 (0.4%)	7 (1.6%)	14 (1.1%)	0.114	0.162	0.00338	0.00561
Pelvic Inflammatory Disease								
Inflammation Only	0 (0%)	0 (0%)	1 (0.2%)	1 (0.1%)	-	0.137	0.00791	0.011
Tubo-ovarian abscess	0 (0%)	0 (0%)	1 (0.2%)	1 (0.1%)				

Various operation details from our study were found to be significant. Total operation time, length of stay, and days from operation to discharge were significant across all comparisons (Table 2). The abdominal approach had the longest total operation time (204.92 minutes), length of hospital stay (1.9663 days), and days from operation to discharge (1.9571 days) among all three operative techniques. The lengths of the intraperitoneal approach followed and the extraperitoneal approach after that. The number of patients undergoing a concomitant procedure and the number of patients classified as inpatient also differed significantly between the three surgical approaches, with the vaginal extraperitoneal approach having a higher number of concomitant procedures (376 (71.8%)) and the abdominal approach having a higher number of patients classified as inpatient (267 (81.9%)) (Table 3). This point was further confirmed with the univariate and multivariate analysis with six controls and 14 controls respectively, demonstrating them all as significant factors (Table 4, p < 0.001).

**Table 2:** Operation details Numerical variables have the mean outside the parenthesis and the standard deviation within the parenthesis. Categorical variables are expressed as their number, followed by the percentage within the parenthesis. For numeric variables, analysis of variance (ANOVA) was employed. For categorical variables, both the chi-square test and Fisher's exact test were utilized.

Operation Details	Abdominal (N=326)	Extraperitoneal (N=524)	Intraperitoneal (N=425)	Total Cohort (N= 1275)	P-value (Abdominal vs Extraperitoneal)	P-value (Abdominal vs Intraperitoneal)	P-value (Extraperitoneal vs Intraperitoneal)	P-value (all three)
Total Operation Time (min); mean (SD)	204.92 (109.74)	120.75 (60.536)	150.86 (67.423)	152.31 (84.899)	<0.001	<0.001	<0.001	<0.001
Length of Total Hospital Stay (days); mean (SD)	1.9663 (1.3343)	0.84160 (0.78933)	1.0847 (0.85361)	1.2102 (1.0770)	<0.001	<0.001	<0.001	<0.001
Days from Operation to Discharge; mean (SD)	1.9571 (1.3236)	0.83969 (0.79016)	1.0847 (0.85361)	1.2071 (1.0725)	<0.001	<0.001	<0.001	<0.001
Prolonged Operation Time	186 (57.1%)	118 (22.5%)	190 (44.7%)	494 (38.7%)	<0.001	<0.001	<0.001	<0.001
Prolonged Total Hospital Stay	203 (62.3%)	50 (9.5%)	94 (22.1%)	347 (27.2%)	<0.001	<0.001	<0.001	<0.001
Prolonged Days from Operation to Discharge	203 (62.3%)	50 (9.5%)	94 (22.1%)	347 (27.2%)	<0.001	<0.001	<0.001	<0.001
Concomitant Procedure	263 (80.7%)	376 (71.8%)	356 (83.8%)	995 (78.0%)	0.00444	0.314	<0.001	<0.001
Inpatient	267 (81.9%)	149 (28.4%)	170 (40.0%)	586 (46.0%)	<0.001	<0.001	<0.001	<0.001
Discharge Home	323 (99.1%)	522 (99.6%)	422 (99.3%)	1267 (99.4%)	0.591	1	0.814	0.607
Wound Classification								
Class 1: Clean	10 (3.1%)	16 (3.1%)	6 (1.4%)	32 (2.5%)	1	0.193	0.146	0.208
Class 2: Clean/Contaminated	316 (96.9%)	507 (96.8%)	413 (97.2%)	1236 (96.9%)	1	1	0.853	0.932
Class 3: Contaminated	0 (0%)	1 (0.2%)	4 (0.9%)	5 (0.4%)	1	0.211	0.179	0.116
Class 4: Dirty/Infected	0 (0%)	0 (0%)	2 (0.5%)	2 (0.2%)	-	0.599	0.2	0.176
ASA Classification								
Class 1	307 (94.2%)	497 (94.8%)	397 (93.4%)	1201 (94.2%)	0.789	0.784	0.423	0.642
Class 2	99 (30.4%)	217 (41.4%)	157 (36.9%)	473 (37.1%)	0.00154	0.0709	0.182	0.00522
Class 3	246 (75.5%)	338 (64.5%)	297 (69.9%)	881 (69.1%)	0.00106	0.107	0.0926	0.00321
Class 4	326 (100%)	521 (99.4%)	424 (99.8%)	1271 (99.7%)	0.439	1	0.769	0.328
Anesthesia								
General	323 (99.1%)	499 (95.2%)	414 (97.4%)	1236 (96.9%)	0.00422	0.161	0.114	0.00518
Spinal	2 (0.6%)	18 (3.4%)	8 (1.9%)	28 (2.2%)	0.0161	0.237	0.209	0.0208
MAC/IV Sedation	1 (0.3%)	6 (1.1%)	1 (0.2%)	8 (0.6%)	0.26	1	0.138	0.185
Epidural	0 (0%)	1 (0.2%)	0 (0%)	1 (0.1%)	1	-	1	1
Regional	0 (0%)	0 (0%)	2 (0.5%)	2 (0.2%)	-	0.508	0.2	0.176
Physician								
Gynecology	296 (90.8%)	487 (92.9%)	413 (97.2%)	1196 (93.8%)	0.319	<0.001	0.00534	<0.001
Obstetrician-Gynecologist	31 (9.5%)	87 (16.6%)	56 (13.2%)	174 (13.6%)	<0.001	0.0421	0.228	0.00289
Urogynecologist	232 (71.2%)	303 (57.8%)	247 (58.1%)	782 (61.3%)	0.00356	0.0434	0.443	0.0105
Gynecologic Oncologist	4 (1.2%)	5 (1.0%)	15 (3.5%)	24 (1.9%)	1	0.0449	0.00925	0.00495
Urology	28 (8.6%)	30 (5.7%)	10 (2.4%)	68 (5.3%)	0.142	<0.001	0.016	<0.001
Plastics	1 (0.3%)	1 (0.2%)	0 (0%)	2 (0.2%)	1	0.434	1	0.726
General Surgery	1 (0.3%)	6 (1.1%)	2 (0.5%)	9 (0.7%)	0.26	1	0.308	0.402

**Table 3:** Complications Numerical variables have the mean outside the parenthesis and the standard deviation within the parenthesis. Categorical variables are expressed as their number, followed by the percentage within the parenthesis. For numeric variables, analysis of variance (ANOVA) was employed. For categorical variables, both the chi-square test and Fisher's exact test were utilized.

Complications	Abdominal (N=326)	Extraperitoneal (N=524)	Intraperitoneal (N=425)	Total Cohort (N=1275)	P-value (Abdominal vs Extraperitoneal)	P-value (Abdominal vs Intraperitoneal)	P-value (Extraperitoneal vs Intrapertioneal)	P-value (all three)
Major Complications	14 (4.3%)	25 (4.8%)	10 (2.4%)	49 (3.8%)	0.877	0.197	0.0731	0.138
Superficial Incisional SSI	9 (2.8%)	5 (1.0%)	4 (0.9%)	18 (1.4%)	0.0827	0.107	1	0.0835
Organ/Space SSI	0 (0%)	4 (0.8%)	2 (0.5%)	6 (0.5%)	0.304	0.508	0.697	0.342
Pulmonary Embolism	1 (0.3%)	1 (0.2%)	0 (0%)	2 (0.2%)	1	0.434	1	0.726
Unplanned Intubation	0 (0%)	0 (0%)	1 (0.2%)	1 (0.1%)	-	1	0.448	0.589
On Ventilator > 48 Hours	0 (0%)	0 (0%)	1 (0.2%)	1 (0.1%)	-	1	0.448	0.589
Acute Renal Failure	0 (0%)	0 (0%)	1 (0.2%)	1 (0.1%)	-	1	0.448	0.589
Myocardial Infarction	0 (0%)	0 (0%)	2 (0.5%)	2 (0.2%)	-	0.508	0.448	0.176
DVT/Thrombophlebitis	1 (0.3%)	1 (0.2%)	0 (0%)	2 (0.2%)	1	0.434	1	0.726
Sepsis	0 (0%)	1 (0.2%)	0 (0%)	1 (0.1%)	1	-	1	1
Septic Shock	0 (0%)	1 (0.2%)	1 (0.2%)	2 (0.2%)	1	1	1	1
Wound Disruption	0 (0%)	0 (0%)	1 (0.2%)	1 (0.1%)	-	1	0.448	0.589
Ureteral Obstruction	0 (0%)	1 (0.2%)	1 (0.2%)	2 (0.2%)	1	1	1	1
Prolonged Urinary Retention	0 (0%)	8 (1.5%)	1 (0.2%)	9 (0.7%)	0.0267	1	0.0474	0.0162
Ureteral Fistula	1 (0.3%)	0 (0%)	0 (0%)	1 (0.1%)	0.384	0.434	-	0.256
Unplanned Reoperation	6 (1.8%)	7 (1.3%)	5 (1.2%)	18 (1.4%)	0.576	0.545	1	0.769
Any Readmission	11 (3.4%)	14 (2.7%)	5 (1.2%)	30 (2.4%)	0.703	0.0699	0.161	0.118
Minor Complications	28 (8.6%)	30 (5.7%)	22 (5.2%)	80 (6.3%)	0.142	0.087	0.821	0.128
Urinary Tract Infection	16 (4.9%)	25 (4.8%)	16 (3.8%)	57 (4.5%)	1	0.557	0.55	0.686
Superficial Incisional SSI	9 (2.8%)	5 (1.0%)	4 (0.9%)	18 (1.4%)	0.0827	0.107	1	0.0835
Bleeding Transfusions	5 (1.5%)	1 (0.2%)	4 (0.9%)	10 (0.8%)	0.0334	0.512	0.179	0.069

**Table 4:** Logistic regression model for 30-day perioperative adverse events following colpopexy 95% Confidence Interval (CI) is expressed as a range. P-value was obtained using logistic regression. The code "Ref" here refers to which surgical approach was selected as the reference for the analysis.

Items	Univariate Analysis			Multivariate Analysis		
Surgical Approach	Odds Ratio	95% CI	P-value	Odds Ratio	95% CI	P-value
Abdominal	Ref	Ref	Ref	Ref	Ref	Ref
Extraperitoneal	0.98	0.86–1.12	0.815	0.99	0.86–1.13	0.84
Intraperitoneal	0.95	0.83–1.10	0.509	0.96	0.83–1.10	0.519
Surgical Approach	Odds Ratio	95% CI	P-value	Odds Ratio	95% CI	P-value
Extraperitoneal	Ref	Ref	Ref	Ref	Ref	Ref
Abdominal	1.02	0.89–1.16	0.815	1.01	0.89–1.16	0.84
Intraperitoneal	0.97	0.86–1.10	0.624	0.97	0.85–1.10	0.614
Surgical Approach	Odds Ratio	95% CI	P-value	Odds Ratio	95% CI	P-value
Intraperitoneal	Ref	Ref	Ref	Ref	Ref	Ref
Abdominal	1.05	0.91–1.20	0.509	1.05	0.91–1.20	0.519
Extraperitoneal	1.03	0.91–1.17	0.624	1.03	0.91–1.17	0.614

Among the cohort, 129 (10.1%) experienced complications from the procedure, with no significant difference in the number of major or minor complications experienced between the three surgical approaches studied. Amongst the major complications of unplanned intubation, patient placed on a ventilator for greater than 48 hours, acute renal failure, myocardial infarction, and wound disruption, there was a significant difference between the abdominal and vaginal extraperitoneal surgical approach (p < 0.001). There was also a significant difference between the abdominal and intraperitoneal approach for the complication of sepsis (p < 0.001; Table 4). All three surgical approaches were significantly different for the major complications of ureteral obstruction, prolonged urinary retention, and ureteral fistula (p < 0.001; Table 5).

**Table 5:** Logistic regression of complications 95% Confidence interval (CI) is expressed as a range. P-value was obtained using logistic regression. The code "Ref" here refers to which surgical approach was selected as the reference for the analysis. SSI: superficial surgical site infection

Variable Name	Surgery Type	Univariate Analysis			Multivariate Analysis		
		Odds Ratio	95% CI	p-value	Odds Ratio	95% CI	p-value
Prolonged Total Hospital Stay	Abdominal	Ref	Ref	Ref	Ref	Ref	Ref
	Vaginal Extraperitoneal	0.06	0.04 – 0.09	<0.001	0.07	0.04 – 0.09	<0.001
	Vaginal Intraperitoneal	0.17	0.12 – 0.24	<0.001	0.18	0.13 – 0.25	<0.001
Prolonged Days from Operation to Discharge	Abdominal	Ref	Ref	Ref	Ref	Ref	Ref
	Vaginal Extraperitoneal	0.06	0.04 – 0.09	<0.001	0.07	0.04 – 0.09	<0.001
	Vaginal Intraperitoneal	0.17	0.12 – 0.24	<0.001	0.18	0.13 – 0.25	<0.001
Prolonged Operation Time	Abdominal	Ref	Ref	Ref	Ref	Ref	Ref
	Vaginal Extraperitoneal	0.22	0.16 – 0.29	<0.001	0.19	0.14 – 0.26	<0.001
	Vaginal Intraperitoneal	0.61	0.45 – 0.81	0.001	0.52	0.38 – 0.70	<0.001
Prolonged Urinary Retention	Abdominal	Ref	Ref	Ref	Ref	Ref	Ref
	Vaginal Extraperitoneal	10.75	0.62 – 186.81	0.02	11.53	0.95 – 140.51	0.02
	Vaginal Intraperitoneal	2.31	0.09 – 56.83	0.587	2.74	0.17 – 44.50	0.511
Ureteral Obstruction	Abdominal	Ref	Ref	Ref	Ref	Ref	Ref
	Vaginal Extraperitoneal	1.87	0.08 – 46.07	0.688	2.05	0.19 – 22.06	0.638
	Vaginal Intraperitoneal	2.31	0.09 – 56.83	0.587	2.79	0.25 – 31.29	0.496
Superficial Incisional SSI	Abdominal	Ref	Ref	Ref	Ref	Ref	Ref
	Vaginal Extraperitoneal	0.34	0.10–0.99	0.055	0.33	0.10–0.99	0.055
	Vaginal Intraperitoneal	0.33	0.09–1.04	0.071	0.31	0.08–1.00	0.062
Ureteral Fistula	Abdominal	Ref	Ref	Ref	Ref	Ref	Ref
	Vaginal Extraperitoneal	0.21	0.01–5.09	0.292	0.22	0.02–2.32	0.387
	Vaginal Intraperitoneal	0.25	0.01–6.28	0.364	0.28	0.03–2.61	0.376
Any Readmission	Abdominal	Ref	Ref	Ref	Ref	Ref	Ref
	Vaginal Extraperitoneal	0.79	0.35–1.79	0.556	0.85	0.37–1.99	0.703
	Vaginal Intraperitoneal	0.34	0.11–0.95	0.048	0.35	0.11–0.97	0.053
Unplanned Reoperation	Abdominal	Ref	Ref	Ref	Ref	Ref	Ref
	Vaginal Extraperitoneal	0.72	0.24–2.26	0.562	0.86	0.28–2.77	0.797
	Vaginal Intraperitoneal	0.63	0.18–2.13	0.457	0.73	0.21–2.47	0.61
Organ/Space SSI	Abdominal	Ref	Ref	Ref	Ref	Ref	Ref
	Vaginal Extraperitoneal	5.65	0.30–105.20	0.15	5.84	0.43–79.68	0.147
	Vaginal Intraperitoneal	3.85	0.18–80.57	0.322	4.37	0.29–65.19	0.275

In addition to our main findings, we also found that, in patients with a prior history of pelvic and abdominal operations, the vaginal extraperitoneal approach was most commonly used. Out of 524 patients in the vaginal extraperitoneal group, 412 (78.6%) women had a history of a prior pelvic operation (p < 0.001), and 199 (38.0%) women had a history of a prior abdominal operation (p = 0.00444). Our data showed that the highest estimated probability of both morbidity and mortality was found in women who underwent the abdominal approach (0.0013301 and 0.063557, respectively), with a significant difference among the three groups (p < 0.001 and p < 0.001, respectively). The data showed an increase in patients present for a concomitant procedure that underwent a vaginal extraperitoneal approach (p < 0.001). Women in the inpatient service showed a higher rate of the abdominal approach being used in comparison to the other approaches (p < 0.001). In terms of postoperative complications, our data showed a higher rate of prolonged urinary retention in women that underwent the vaginal extraperitoneal approach (8 (1.5%), p < 0.001). Only one patient out of 425 patients (0.2%) in the vaginal intraperitoneal group and no patients in the abdominal approach group experienced prolonged urinary retention. There was an equal rate of one patient (0.2%) that experienced ureteral obstruction postoperatively in both the vaginal extraperitoneal and vaginal intraperitoneal groups, both significantly different than the abdominal group given that there were no patients with a ureteral obstruction (p < 0.001). There was one case out of 236 cases (1 (0.3%), p < 0.001) that used the abdominal approach that developed a ureteral fistula postoperatively.

## Discussion

In this retrospective study, we analyzed the differences between the three approaches to address symptomatic pelvic organ prolapse with sacrocolpopexy. Overall, we found that there were few significant complication rates between abdominal, vaginal extraperitoneal, and vaginal intraperitoneal approaches. The extraperitoneal approach had an increased number of patients with prolonged urinary retention. Furthermore, between the abdominal and extraperitoneal approaches, complications were only significant with the abdominal approach with an increased occurrence of bleeding transfusions.

Considering the extent of invasiveness associated with the abdominal approach, it is logical to anticipate a prolonged postoperative recovery period and increased operation time when compared to both of the vaginal approaches. Concurrently, our data support this statement, which is shown by the significant decrease in the odds ratio when the vaginal approaches are compared to the abdominal approach for these variables. Linder et al. evaluated prolonged hospitalization and 30-day complication rates, amongst other factors, in 4,362 women who underwent an abdominal sacrocolpopexy and minimally invasive laparoscopic-/robotic sacrocolpopexy. The abdominal approach demonstrated a prolonged hospitalization after the procedure in comparison to the minimally invasive technique (p < 0.001) [9]. Our study also echoes Dingqian et al., who explained that the extraperitoneal approach is performed entirely outside of the peritoneum, so there is less restriction by factors such as obesity, abdominal adhesion, occlusion of bowel position, and other surgical obstacles. The avoidance of these obstacles allows for increased postoperative pain relief based on the principle of Enhanced Recovery After Surgery (ERAS) [10].

However, there are differences in the findings of our study in comparison to Bretschneider et al. in regards to the rate of UTI complications following either a vaginal extraperitoneal or vaginal intraperitoneal approach. Per Bretschneider et al., one in 10 women undergoing vaginal colpopexy experienced a postoperative complication, with UTI being the most common [11]. Although having a UTI was not a significant complication found in our study, the extraperitoneal approach did show a significant amount of people who had prolonged urinary retention. In fact, our data also show that the extraperitoneal vaginal approach cohort had a significantly older age compared to the other two approaches. Together, these data support the results also found by Yune et al., which demonstrated older age as a risk factor for postoperative urinary retention in those patients that underwent transvaginal vs. robotic colpopexy [12].

Another statistically significant finding in our study showed that between the two vaginal approaches, more people using the extraperitoneal approach had prior pelvic operations, as well as concomitant procedures than those using the intraperitoneal vaginal approach. These findings demonstrate that pelvic organ prolapse is commonly associated with other surgical operations, suggesting that a sacrocolpopexy is the secondary procedure. Therefore, close attention should be given to which approach is chosen when performing the concomitant or primary procedure. For example, several studies have explored whether abdominal or laparoscopic sacrocolpopexy has higher complication rates when performed with a hysterectomy. Although one study could not find complication rates that were significantly different between the abdominal or laparoscopic approaches, they did find that the abdominal approach had more severe complications [13]. Our study does not include data on laparoscopic sacrocolpopexy but does include less invasive techniques, such as the vaginal approaches, which are compared to the abdominal sacrocolpopexy approach. However, another study revealed that the extraperitoneal approach provides shorter operative times than the intraperitoneal approach when there is a history of prior abdominal surgeries or pelvic disease, but, as a whole, both approaches had lower rates of perioperative complications [14]. While our study does not aim to prefer one approach over the other, this aspect is discussed to highlight the possible complications between the approaches when a prior surgical history is present or if a concomitant procedure must be performed.

Our study has several strengths, including the use of the ACS NSQIP. This database is the first nationally validated program with the participation of over 3,400 hospitals and facilities [15]. To our knowledge, we are the first to utilize this database regarding peri- and postoperative complications in abdominal, vaginal extraperitoneal, and intraperitoneal colpopexy.

Limitations for our study begin with the retrospective design of our study that does not include more recent data points from 2021 and 2022. Additionally, it is important to understand when a sacrocolpopexy is an indicated procedure. Given the significant rate of concomitant procedures in patients undergoing a sacrocolpopexy in our study, we must consider this as a limitation due to the natural increase in operation time, total hospital stay, and total days from operation to discharge. Additionally, the degree of prolapse was not explored in the collection of our data.

## Conclusions

In conclusion, this study provides valuable insights into the three surgical approaches for sacrocolpopexy. Although few significant complications were found between the three approaches, the data found significant in regards to operation details encourage future studies to explore what other procedures are performed concomitantly with sacrocolpopexy. Given the limitations of our study, including a larger sample size and degree of prolapse would enhance the generalizability of our findings. Ultimately, we hope that our work will encourage further investigation and discussion and aid in the surgical decision-making of POP.
